# Utilization of Birch Bark as an Eco-Friendly Filler in Urea-Formaldehyde Adhesives for Plywood Manufacturing

**DOI:** 10.3390/polym13040511

**Published:** 2021-02-08

**Authors:** Roman Réh, Ľuboš Krišťák, Ján Sedliačik, Pavlo Bekhta, Monika Božiková, Daniela Kunecová, Vlasta Vozárová, Eugenia Mariana Tudor, Petar Antov, Viktor Savov

**Affiliations:** 1 Faculty of Wood Sciences and Technology, Technical University in Zvolen, 960 01 Zvolen, Slovakia; reh@tuzvo.sk (R.R.); sedliacik@tuzvo.sk (J.S.); 2 Department of Wood-Based Composites, Cellulose and Paper, Ukrainian National Forestry University, 79057 Lviv, Ukraine; bekhta@nltu.edu.ua; 3 Faculty of Engineering, Slovak University of Agriculture in Nitra, 949 76 Nitra, Slovakia; monika.bozikova@uniag.sk (M.B.); daniela.kunecova@uniag.sk (D.K.); vlasta.vozarova@uniag.sk (V.V.); 4 Forest Products Technology and Timber Construction Department, Salzburg University of Applied Sciences, 5431 Kuchl, Austria; Eugenia.tudor@fh-salzburg.ac.at; 5 Faculty of Wood Engineering, Transilvania University of Brasov, 500036 Brasov, Romania; 6 Faculty of Forest Industry, University of Forestry, 1797 Sofia, Bulgaria; victor_savov@ltu.bg

**Keywords:** adhesive fillers, ground birch bark, beech plywood, eco-friendly fillers, UF resin, formaldehyde emission

## Abstract

The potential of using ground birch (*Betula verrucosa* Ehrh.) bark as an eco-friendly additive in urea-formaldehyde (UF) adhesives for plywood manufacturing was investigated in this work. Five-ply plywood panels were fabricated in the laboratory from beech (*Fagus sylvatica* L.) veneers bonded with UF adhesive formulations comprising three addition levels of birch bark (BB) as a filler (10%, 15%, and 20%). Two UF resin formulations filled with 10% and 20% wheat flour (WF) were used as reference samples. The mechanical properties (bending strength, modulus of elasticity and shear strength) of the laboratory-fabricated plywood panels, bonded with the addition of BB in the adhesive mixture, were evaluated and compared with the European standard requirements (EN 310 and EN 314-2). The mechanical strength of the plywood with the addition of BB in the adhesive mixture is acceptable and met the European standard requirements. Markedly, the positive effect of BB in the UF adhesive mixture on the reduction of formaldehyde emission from plywood panels was also confirmed. Initially, the most significant decrease in formaldehyde release (up to 14%) was measured for the plywood sample, produced with 15% BB. After four weeks, the decrease in formaldehyde was estimated up to 51% for the sample manufactured with 20% BB. The performed differential scanning calorimetry (DSC), thermal gravimetric analysis (TGA), and derivative thermogravimetry (DTG), also confirmed the findings of the study. As this research demonstrated, BB as a waste or by-product of wood processing industry, can be efficiently utilized as an environmentally friendly, inexpensive alternative to WF as a filler in UF adhesive formulations for plywood manufacturing.

## 1. Introduction

Urea-formaldehyde (UF) adhesives are by far the most widely used thermosetting resins for manufacturing plywood and other types of interior-grade wood-based composites [1,2,3], accounting for nearly 85% of the total amino resins produced worldwide with an approximate annual volume of 11 million tons/year [4,5,6]. The wide industrial application of these resins in the production of wood-based composites is due to their significantly strength performance, chemical versatility, short press times, low curing temperatures, solubility in water, colorless glue line, ease of handling, and a relatively low cost [7,8,9,10]. However, the main drawbacks of UF resins are the significantly lower water resistance, compared to phenolic and melamine-formaldehyde resins, and emission of formaldehyde which is associated with environmental problems [11] and human health-related hazards [12,13,14].

UF adhesives create the high bonding strength of veneers and largely determine the physical, mechanical and other properties of plywood [15,16]. However, they are consisted not only of pure oligomers, but also of different additives such as extenders, fillers, etc., and therefore, the resulting adhesive effect is also dependent on the proportion of other components of the UF adhesive mixture used [2,17,18].

Fillers are essential components of UF adhesives, added to glue mixtures mostly in a solid state [19,20] for various reasons—to reduce the plywood production costs, to restrict the undesired fluidity or excessive penetration of adhesive into veneers, and to build a solid structure of the cured adhesive composition [3,15,21].

Fillers can also play an important task in the reduction of free formaldehyde release from UF adhesive mixtures, especially in the production of plywood panels [20,22,23,24,25,26,27]. According to our research [28,29], and other research [19,20,30], it suggests that the ground bark of some tree species, applied as a filler with UF resins in plywood manufacture, leads to significant decrease in free formaldehyde release. The type and the amount of filler in the adhesive mixture significantly affect the adhesive performance. The choice of filler depends on the resin and targeted application, making fillers one of the most important components of adhesive systems used in the production of plywood [15,18,19,31,32].

Commonly used adhesive fillers are nonvolatile, organic substances, such as wheat or rye flour, soybean powder, rice husk, wood powder, bark powder or other lignocellulosic wastes, such as palm kernel, coconut shells, starch material, etc. [27,31]. In addition, inorganic materials such as metal powders, metal oxides, and minerals (clay, sepiolite, wollastonite) have also been used as adhesive fillers, typically to improve compression strength and dimensional stability [15,30,33,34].

This publication is focused only on fillers made from birch bark (*Betula verrucosa* Ehrh.). Birch is a high-quality raw material for plywood processing which has been extensively used in plywood industry [35,36]. If birch is used for manufacturing veneer-based products by wood peeling, it must be debarked and therefore it is assumed that considerable amounts of bark are available in plywood mills which need to be further utilized [37,38]. Its use as a filler in UF adhesive formulations, among other utilization purposes, is possible and recommended.

Birch bark (BB) of mature trunks and branches is creamy to silvery white, smooth, it peels off as long strands; lenticels are dark, horizontally expanded [39,40]. BB was successfully utilized even in the past, and at present extensive research is being carried out in the field of pharmaceutical and cosmetic industry. BB extracts have been widely used in modern cosmetics and have a potential usage as dietary supplements [38,41].

Chemical composition of BB has gained a significant scientific interest because it contains higher concentrations of extractives than birch wood. The concentrations of individual compounds are quite low in BB (less than 1%) except for betulinol (up to 12.7%). Outer BB contains about 40% extractives, 45% suberin, 9% lignin, 4% hemicelluloses and 2% cellulose. The extractives mainly comprise different triterpenoids, especially betulin and its derivatives, accounting for as much as 30% of the dry weight. The outer BB contains only about 2 mg·g^−1^ of phenolics, mainly comprising esterified hydroxycinnamic acids, whereas the inner bark has very high phenolics contents [42,43].

Considerable efforts have been made by researchers to decrease or eliminate the harmful formaldehyde emissions from UF-bonded wood-based panels, including plywood. This can be done through several procedures that can be truly effective—during the manufacturing process or by post-treatment of wood-based composites. Major approaches to minimize formaldehyde emissions include the following [20,26]: (a) reduction of formaldehyde content in resin formulation [24], (b) the use of scavengers [44,45,46,47,48] or other compounds that reduce the free formaldehyde [49,50,51], (c) posttreatment of the wood-based products or surface treatment with paints, lacquers, veneers, and papers [51,52,53], and (d) the use of alternative adhesive, bio-based adhesive systems [54,55,56,57,58,59,60].

A possible way to reduce formaldehyde emissions from plywood panels is to use scavengers, to replace technical flour using tree bark as a sustainable, inexpensive filler for UF adhesive systems [37,38,45,46,49]. Currently, bark is burned in plywood mills, and its necessary production is considered as a load, therefore the incorporation of bark into adhesive mixtures would relieve the plywood producers from great amount of by-products and waste. This will also result in reduced production costs, since the purchase of food flour as a filler would not be necessary, as the bark comes from its own unused material stocks in plywood mills [19,20,28,29].

Therefore, the aim of this research work was to investigate the potential of using ground BB as an eco-friendly filler in UF adhesive in plywood manufacture and determine its effects on the physical, mechanical, and thermal properties of plywood, as well as on the free formaldehyde emission of panels.

## 2. Materials and Methods

### 2.1. Bark Collection, Drying, and Grinding

BB was manually removed from a trunk harvested near Zvolen, Slovakia (SK) for the purposes of this research. The birch log was stored outdoors for several weeks under normal climatic conditions as in the common factory practice. The debarking of the birch trunk was relatively easy and it was performed with hand tools (axe, double-edged knife). The birch log was about 40-year-old, so the bark was hard and cracked as some places.

Fragments of BB measuring about 50 × 20–30 mm in a volume of about 30 L were first washed with distilled water to remove dirt and mineral particles, and then dried in bulk for about 3 weeks in a heated room at the temperature of 18 °C. The bark reached moisture content (MC) of approximately 14%. Subsequently, the bark was dried in a laboratory oven at 103 ± 2 °C to a MC of 3 ± 1%. After reaching the assumed MC, the BB was ground by a semi-operational impact cross mill (prototype, Technical University in Zvolen, SK), and then by a fine hand grinder to obtain the required size of the ground bark fraction. The bark grinding equipment directly affects the quality, quantity and geometry of the bark [61]. Bark was sieved with mechanical sieve shaker (prototype, Technical University in Zvolen, SK) to obtain a dimensional fraction fallen through the screen with a mesh size of 0.125 mm which was determined by the authors as the most effective in a previously conducted research [28,29]. A total of 1.2 kg of ground BB was produced and only the finest fraction of bark with grains equal or smaller than 0.125 mm was added to the adhesive mixture. BB was stored in plastic bags to protect it from moisture increase. The collected material was used to modify UF resin for plywood manufacturing.

### 2.2. Chemical Composition of BB Flour

Proper bark valorization requires a detailed analysis of its chemical composition. The chemical composition of BB flour, including its main components such as holocellulose, lignin, ash, and extractives, and carbohydrate composition, is summarized in Table 1 and Table 2, respectively.

Ground BB with its acidic nature and alkaline buffering capacity is suitable to obtain reasonable pressing time in plywood manufacturing, especially when using pH-depended resins like UF [63,64]. As known, UF resins are acid-catalyzed adhesives and need an acidic environment to cure [65,66]. In this respect, birch with pH values ranging from 3.2 to 5.0 depending on the harvesting site [67] is a promising material for plywood manufacturing. The pH value of BB is expected to provide an appropriate environment for curing of UF resin during the fabrication of plywood which might slightly affect the strength properties of the finished panels.

BB has a high content of lignin and adding it to the adhesive composition should reduce the content of free formaldehyde in the finished plywood panels. Previous research showed that lignin can absorb up to 15% of formaldehyde based on its weight [68]. Most of the binding of formaldehyde by lignin is due to the Tollens reaction, Prins reaction or Lederer–Manasse reaction [69]. Formaldehyde also reacts with extractives in bark, mostly with tannins which was confirmed in case of plywood, particleboard and bark boards [28,70,71].

### 2.3. Veneers

Wood raw material—beech (*Fagus sylvatica* L.) from the central region of Poľana Mountains in Slovakia was used for peeling. Beech veneers were made by centric peeling process using a 4-feet lathe KSB (Královopolská strojírna Brno, Czechia) at the Technical University in Zvolen, Slovakia. The veneer sheets with the dimensions of 500 mm × 500 mm without visible defects were prepared for the experiments. Average thickness of veneers was 1.30 mm, and the average density was 628 kg·m^−3^. Their MC after drying and conditioning was 6 ± 1%. Test groups of five-ply plywood panels made of beech veneers glued together with adhesive mixture of different composition were fabricated under laboratory conditions.

### 2.4. Adhesive and Additives

A commercially available UF resin Kronores CB 1100 F (Diakol Strážske s.r.o., Strážske, Slovakia) with the solid content of 67.1%, viscosity of 460 mPa·s, condensation time of 55 s, and a pH value of 8.6 was used to bond veneers. Ammonium nitrate NH_4_NO_3_ (47%) was added to the adhesive mixture as a hardener. Commercial technical wheat flour (WF) was used as the control filler to evaluate the suitability of BB for plywood manufacturing, since it is the most widely used filler in plywood industry.

### 2.5. Adhesive Mixture Preparation

The ammonium nitrate hardener was added at a ratio of 10 weight parts (wp) per 100 wp of adhesive according to the common industrial formulations. UF adhesive with 10 and 20 wp per 100 wp of WF based on liquid UF resin were used as reference samples. They were labeled as Ref10 and Ref20, respectively. No water was added. The amount of added BB flour was 10, 15, and 20 wp per 100 wp of liquid UF resin. They were labeled as BB10, BB15, and BB20, respectively (Table 3). Viscosity was measured using the rotary viscometer at 20 °C using standard test methods for viscosity of adhesives.

### 2.6. Plywood Panel Manufacturing

Adhesive mixtures were applied to the veneers in laboratory conditions using a hand roller to form a uniform adhesive layer. The amount of adhesive spread was calculated according to recommendations for UF adhesive mixtures per 1 m^2^ (180 g·m^−2^) based on the wet mass. The UF adhesive mixture was applied onto one side of each veneer sheet (except for the last one). When composing veneers, the fibers of the neighboring veneers were at 90° angle (veneer sheets laid up tight/loose) in accordance with the standard EN 636:2015 [72].

The pressing process was carried out using the CBJ single opening laboratory electric heated hydraulic press (CBJ250, TOS, Rakovník, Czechia). The pressing temperature used was 105 °C; the specific pressing pressure was 1.8 MPa. The pressing time was 336 s and it was calculated as the sum of the basic pressing time for UF adhesives recommended by the producer and the corresponding thickness of the pressed veneers.

Groups of five-ply plywood made of beech veneers glued together with adhesive mixture of different composition were formed under laboratory conditions. Five plywood panels were made for each experimental condition. Five-ply plywood panels of 500 mm × 500 mm were made to determine the bending strength (MOR), modulus of elasticity (MOE), shear strength and formaldehyde release of plywood panels.

### 2.7. Plywood Panel Testing

After pressing, all plywood panels were conditioned at 20 ± 2 °C temperature and 65 ± 5% relative humidity for 4 weeks until constant mass was achieved. The MC after conditioning was calculated according to the standard ISO 13061-1:2014 [73]. After conditioning, plywood was cut into exact test samples according to the EN 326-1 standard requirements [74].

MOR and MOE of plywood panels were determined according to the standard EN 310 [75]. The crosshead speed used was 1.0 mm·min^−1^, the load cell configuration was 5 kN. The shear strength was measured according to the EN 314-1 [76] and classified according to the EN 314-2 [77] standards after pretreatment for intended use in interior conditions. Testing samples were immersed in water for 24 h at 20 ± 3 °C. Twelve samples were used for each variant for testing the MOR, MOE and shear strength of the laboratory-fabricated plywood panels. MOR, MOE and shear strength tests were carried out using a TIRA 2200 Heckert Testing Machine (Schalkau, Germany) for all adhesive mixtures (Ref10, Ref20, BB10, BB15, and BB20).

### 2.8. Formaldehyde Emission from Plywood Panels

The free formaldehyde emission was measured using the desiccator method according to the standard EN-ISO 12460-4 [78]. The test determines the quantity of formaldehyde emitted from plywood samples and absorbed in a specified volume of distilled water during 24 h in a glass desiccator. Ten test pieces from each type of plywood were prepared with dimensions of 150 ± 1 mm × 50 ± 1 mm (length × width) for a total surface area of 1735 cm^2^. They were then placed in a desiccator with an enclosed volume of 11 L with a glass-crystallizing dish containing 300 mL of distilled water. Samples were removed from the desiccator after 24 h and the obtained formaldehyde solution was prepared for spectroscopy. To determine the formaldehyde content, 25 mL of the formaldehyde solution from the desiccator was mixed with 25 mL of acetylacetone-ammonium acetate solution in a 100 mL flask. The stoppered flasks were then heated in a water bath at 65 ± 2 °C for 10 min and subsequently cooled to ambient temperature for 60 ± 5 min [20,79].

A UviLine SI 5000 spectrophotometer (SI Analytics, Plains, NY, USA) at 412 nm was used to determine the total formaldehyde content. The formaldehyde content was determined using a calibration curve which was prepared from standard formaldehyde solutions. Formaldehyde emission tests were carried out in duplicate [20,79].

### 2.9. Thermal Analyses DSC, TGA and DTG

The device DSC 1 (Mettler-Toledo GmbH, Greifensee, Switzerland) was used to monitor the curing processes by the differential scanning calorimetry (DSC) method. This device provides accurate and reliable results of measuring the enthalpy depending on the temperature for the selected temperature program. The device provides the capability to monitor endothermic and exothermic processes which correspond to the peaks and changes the shape of the DSC curve [80,81]. For each test, the results were obtained in the form of important parameters such as “onset” temperature (*T*_o_), and temperature of peak reached during the reaction (which is also the temperature when the curing is completed) [82]. It is known that linear resin monomers are converted into crosslinking networks during curing. The cure reactions of resins are very complex because many reactive processes occur simultaneously has been studied by means of differential scanning calorimetry [83,84]. Results commonly depend on several factors such as the size of the fillers, the percentage loading and the dispersion of the particles [80,85].

Thermal gravimetric analysis (TGA) can be used to monitor weight changes in a sample as a function of temperature. The technique is primarily used for studying degradation processes, providing information on thermal oxidative degradation rates and thermal degradation temperatures of polymeric materials. The technique is not particularly sensitive to the changes in adhesives and resins due to different states of cure and this is the main reason it has not been developed further for cure monitoring. According to the shape of the TGA curves, the materials determine the rate of the reaction and the temperature of the decomposition processes. From thermal degradation of materials, we can identify thermal resistance [85,86].

Three types of thermal analyzes were used for the samples, namely DSC, TGA together with derivative thermogravimetry (DTG). DTG as a technique was used for identifying and quantitatively analyzing the chemical composition of substances by observing the thermal behavior of a sample as it is heated.

The temperature methodology of the experiment was set according to the needs of practice. The temperature was increased from 25 °C to 105 °C at a heating rate of 10 °C∙min^−1^. Isothermal part of experiment occurred after reaching 105 °C which lasted 10 min. Upon completion the temperature was raised to 110 °C with a heating rate of 15 °C∙min^−1^. After achieving the temperature 110 °C the sample was cooled to an operating temperature of 25 °C. TGA was performed in oxygen atmosphere throughout the experiment. As carrier gas was used nitrogen (purity 99%) at DSC method. Gas flow was for both methods 20 mL∙min^−1^.

The device TGA/DSC1 was employed to measure the changes of mass in the samples and to monitor the temperature processes. TGA measurement was realized in alumina crucible with lids and with diameter of 6 mm and length of 4.5 mm. The total volumes of the crucibles were 70 μL and the lids were pierced. Simultaneously with the thermogravimetric analysis, a DSC measurement was performed. The sample was weighed by scales KERN ABT 220-5DM version 1.2 03/2013 (Kern & Sohn GmbH, Balingen, Germany) into a 100 μL aluminum crucible with a diameter of 6 mm and a height of 4 mm.

### 2.10. Statistical Analysis

All the data were analyzed using analysis of variance (ANOVA). The comparison of the means was carried out employing Duncan’s test with a 95% confidence level. The standard deviations were also computed from the data.

## 3. Results and Discussion

### 3.1. Viscosity

Dynamic viscosity (mPa·s) of the adhesive mixture used (UF + hardener + WF/BB flour) has a strong effect on its application to veneers. Different dynamic viscosity values were determined with the different proportions of BB flour addition following the standard test method [87]. Adhesive mixtures with higher viscosity are more difficult to apply on veneers. Adhesive mixtures with lower viscosity could penetrate into veneers better.

The following average values of dynamic viscosity of the used adhesive mixtures were determined (Table 4):

The results show that the addition of BB as a filler in UF adhesive increased the viscosity and reactivity of the UF resin. The adhesive formulation (UF + hardener + BB flour) was more difficult to apply on veneers when the addition of BB was increased to 20%. At all addition levels of BB (10%, 15%, and 20%) the bark powder was dispersed evenly, forming a homogeneous adhesive mixture.

### 3.2. Mechanical Properties

#### 3.2.1. Modulus of Rupture and Modulus of Elasticity of Plywood Panels

The results of the MOR and MOE tests are presented in Table 5.

The determined values of MOE and MOR are equal or slightly higher than the mechanical strengths of commercially produced plywood [88,89]. BB flour acted as a proper filler when the adhesive mixture was spread on veneers, there were no problems with manual application of BB10 and BB15. Applying the BB20 was slightly more demanding due to the increased dynamic viscosity of the adhesive mixture. This experience was also confirmed by other authors [28,29,90] who used higher concentrations (above 15%) of bark from other tree species as a filler.

In case of Ref20 there was a significant decrease in both MOR (104 MPa) and MOE (11,827 MPa). Too much WF bonds almost all the water from the adhesive mixture which leads to very high viscosity. High viscosity of adhesive resulted in a low flow ability of the adhesive and then consequently a poor penetration into wood veneers [91]. Another reason is that increasing the addition of WF in the adhesive reduced the amount of UF resin in the bond line which consequently decreased the adhesion strength of veneers [92].

Comparison of values between BB10 and BB15 showed a significant increase in MOR and MOE properties. The main reason is the high tannin and lignin content in BB. The reaction of tannin with formaldehyde improves cross-linking and leads to reinforcement of adhesives [46]. The high lignin content in BB (approx. 28%) also plays an important role, contributing to form a stable connection with the tannins and improving their reactivity with the hardener, leading to an improvement of adhesion properties [20,93].

Further increase in bark amount (BB20) led to a slight decrease of MOR and MOE values, with a mean of 113 MPa (MOR) and 12,765 MPa (MOE) respectively. On the one hand, there is another increase in tannin and lignin content in resin, that should lead to increase in mechanical properties, on the other hand—high tannin and lignin content results in high reactivity at higher concentrations, which leads to quick viscosity development, and thus to a short pot life of the resin which results in lower intermolecular crosslinking [94,95]. A similar result was also reported by [96]. The authors stated that with increased tannin solution concentration in the UF resin, the MOE values of the panels were slightly decreased. Another reason is analogous to Ref20 and high viscosity of adhesive which results in low flow ability and poor penetration into wood veneers.

#### 3.2.2. Shear Strength of Plywood Panels

The results of the shear strength tests are presented in Table 6.

All shear strength mean values were significantly above the EN 314-2 standard requirement (1 MPa) [77].

Similarly, as in MOR and MOE, in case of Ref20 (2.37 MPa) there was a significant decrease in shear strength (2.22 MPa) compared to Ref10 sample. The high content of WF absorbs most of the water, which participates in forming a three-dimensional crosslinking network structure [31]. An increase in viscosity of UF resin adhesive led also to increase in the bond strength which affected the flow ability of UF resin adhesives, and inhibited the resin penetration into wood veneers [92].

Shear strength increased with an increase in bark concentration. A significant increase of 10.4% was determined in BB10 samples (2.11 MPa) compared to BB15 (2.33 MPa), due to the higher amounts of tannin and lignin in the bark which improves the adhesion properties. Indicator of reactive tannins towards formaldehyde is the Stiasny number, the higher value means the more formaldehyde-condensable tannins are extracted and the more effective the adhesive bonding will be [97].

Even though the increased dynamic viscosity of BB20 the achieved values of shear strength of plywood samples did not reduce. The effect of high lignin and tannin content in the bark is significantly more important than higher viscosity. These results showed that the performance of plywood panels made using UF with BB filler compositions was influenced not only by chemical conditions, such as tannin and lignin content, but are also influenced by the pressing regime applied which was confirmed by another research [18,93]. The authors stated that shear strength increases when the cure time and press temperature increase.

The specific chemical composition of BB in the UF adhesive mixture apparently affected the mechanical properties of plywood to a minimal extent which is also supported by other research works [19,20,31].

### 3.3. Formaldehyde Emission from Plywood Panels

**Figure 1:**
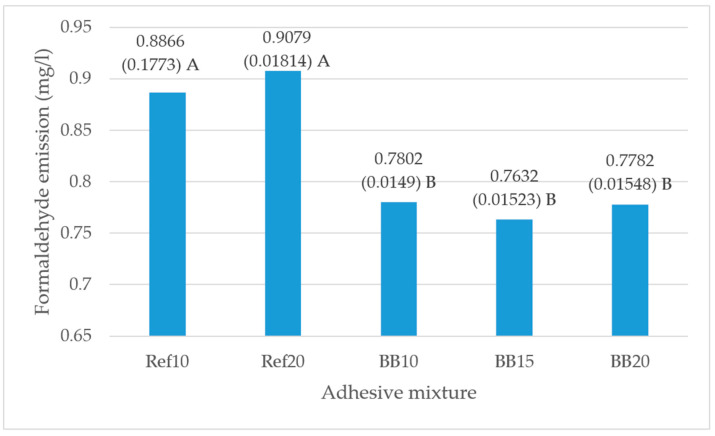
Free formaldehyde emission (mg·L^−1^) in five-layer plywood panels produced with urea-formaldehyde (UF) resin and three addition levels (10%, 15%, and 20%) of BB as adhesive filler. Different letters denote a significant difference. Means followed by the same letter do not statistically differ from each other (*p* ≤ 0.05).

The results obtained for the free formaldehyde emission of the laboratory-fabricated plywood panels, bonded with UF resin and three addition levels of BB as adhesive filler, tested in accordance with the standard EN-ISO 12460-4 (known as desiccator method) [78], are presented in Figure 1. The results clearly demonstrated that the replacement of WF with BB as UF adhesive filler led to a significant decrease in free formaldehyde emissions compared to the reference sample (Ref10). The decrease in formaldehyde emission was estimated to 12% in BB10 and BB20, and to 14% in BB15, respectively. It was also pointed out that the addition of extra 10% of WF in Ref20 compared to Ref10 sample led to slight increase of formaldehyde release (3%).

The reduction in free formaldehyde emissions may be attributed to the high amount of lignin in the chemical composition of tree bark [98,99]. BB is reported to have approximately 50% lignin content [100,101]. Lignin is capable of reacting with formaldehyde in the acidic medium, the formed benzylic alcohols react with a lignin model compound which leads to formation of methylene-linked dimers [102]. In addition, the tannins present in the BB also contributed to the decrease in formaldehyde emission. Due to their phenolic nature, condensed polyflavonoid tannins react with formaldehyde and produce a polymerization through the methylene bridge linkages to reactive positions of the flavonoid molecules [31,86,90,103].

The addition of 15% BB in the adhesive formulation (BB15) resulted in a slight decrease of formaldehyde emission (compared to BB10). Furthermore, the addition of 20% BB resulted in a slight yet still measurable increase of formaldehyde emission. This increase was attributed to the water hydrophilicity of BB as a filler which bonded almost all water from the adhesive mixture, thus leading to increased formaldehyde emission over time. The result of this phenomena is significant decrease of MC in veneers that leads to increase of formaldehyde emission [18].

**Figure 2:**
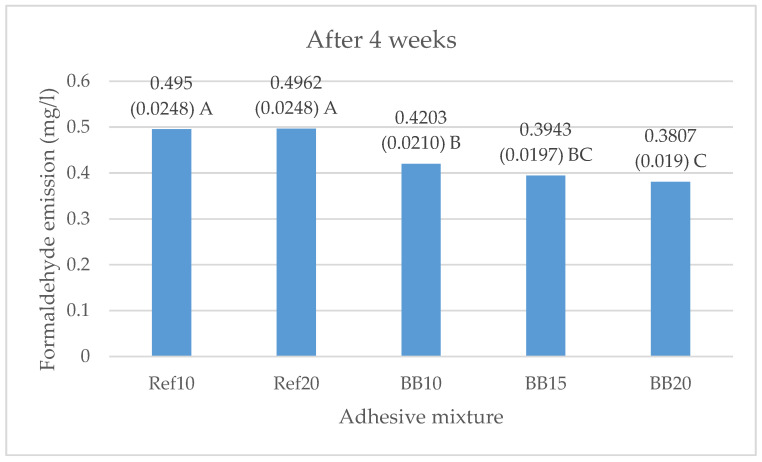
Free formaldehyde emission (mg·L^−1^) in five-layer plywood panels produced with UF resin and three addition levels (10%, 15%, and 20%) of BB as adhesive filler, measured after four weeks. Different letters denote a significant difference. Means followed by the same letter do not statistically differ from each other (*p* ≤ 0.05).

Graphical representation of the effect of BB addition as a filler in UF adhesive on the formaldehyde release of the laboratory-produced plywood panels, measured after 4 weeks of samples conditioning, is presented in Figure 2. Results illustrated that the formaldehyde emissions had a 13% and 20% decrease compared to the reference sample (Ref10), respectively.

As seen in Figure 2, a significantly greater decrease in formaldehyde emission of plywood samples, comprising BB as a filler was measured after four weeks, compared to the tested WF reference samples. The most significant decrease after four weeks (51%, 0.78 mg/L vs. 0.38 mg/L) was recorded for the plywood sample with 20% BB. Results clearly demonstrated the potential of using BB as an organic, eco-friendly filler in UF adhesive which acts as a natural formaldehyde scavenger in plywood panels. As it was shown in a previous research [20], polyphenolic extractives can react with formaldehyde even at ambient temperature. The potential of powdered tree bark to reduce the formaldehyde emission from ply-wood panels was also confirmed by other authors. Research [104] showed up to 48% decrease in formaldehyde emission using 20% Turkish pine (*Pinus brutia* T.) bark, while maintaining the good exploitation properties of the panels. In our previous research [28,29] it was demonstrated that the use of beech bark (10–20%) as a filler in UF adhesive mixture in plywood leads to significant decrease in formaldehyde emission due to tannin content in the bark. Decrease in formaldehyde emission was also confirmed by [20] where adding beech bark (1–5%) in the adhesive composition reduced the free formaldehyde content due to the interaction of formaldehyde with lignin in the bark, and hence, reduced formaldehyde emission of the finished plywood panels. Authors [18] found that the use of flours (10%) from the barks of chestnut and fir trees in the glue mixture decreased formaldehyde emission of plywood panels. Authors concluded that the reason for the low formaldehyde emission values can be explained by the greater amount of polyphenolic extractives in bark than in wood. The effect of BB content in adhesive formulation on the decrease in formaldehyde emissions of plywood panels was also demonstrated by [90]. Results showed that use of 20% BB leads to a 17% decrease in formaldehyde release. The same authors [31] used oak bark powder as filler for MUF adhesive in plywood manufacturing. The formaldehyde emissions, measured initially and after 8 weeks, experienced a 9% and 31% decrease in comparison with the reference variant, respectively. In the study of [105], no change in formaldehyde emission in particleboard was determined in case of addition of 6% acacia bark to the core layer, on the other hand, it was markedly decreased with the ratio of bark from 12 to 25%. Other studies showed formaldehyde decrease using bark in thermal insulation panels [86,98,99,106,107].

### 3.4. Thermal Analysis Methods

#### 3.4.1. DSC

**Figure 3:**
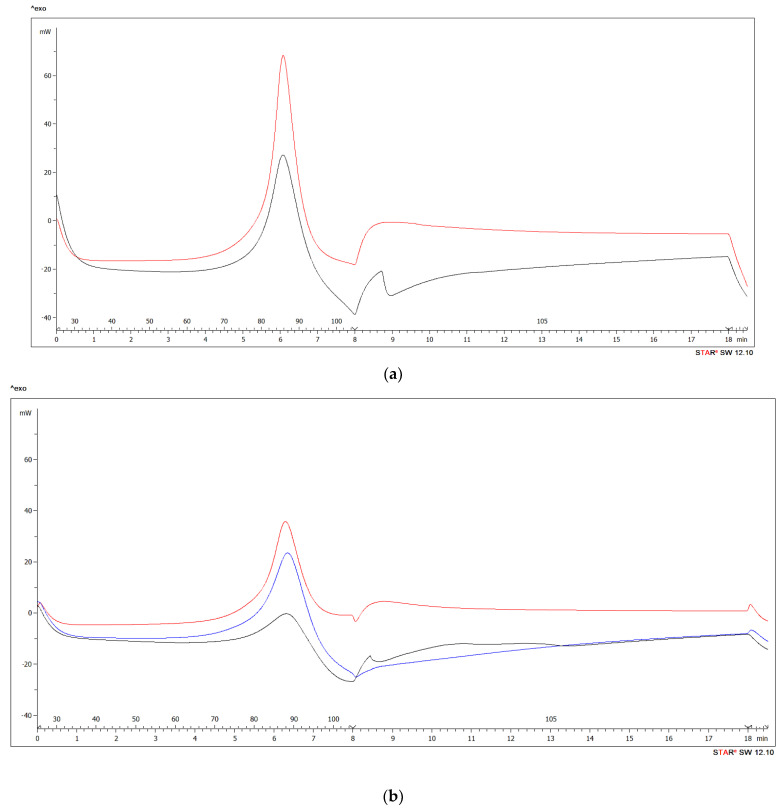
Differential scanning calorimetry (DSC) thermograph for (**a**) wheat flour (WF) at concentration 10% (red) and 20% (black) and (**b**) BB concentration 10% (red), 15% (blue) and 20% (black). Right half of the figure: the isothermal section at the temperature of 105 °C.

DSC methods have proven to be a suitable thermal analysis technique to explain the effect of BB flour addition to UF adhesive mixtures. During tests, the DSC instrument lid was not pierced and it was fixed on a crucible during the investigation. The characterization of curves was carried out with the software STAR^®^ 9.3, based on the recording from the device Mettler-Toledo, and is presented in Figure 3 and Table 7.

The curing reaction started at *T*_onset_ temperature, the so-called initiation temperature. The conversion rate reached a maximum during a dynamic scan of the reaction at *T*_curing_ temperature. Both values were obtained from the DSC curves. The curing reactions of UF resins (both WF reference and BB samples) was represented by an exothermic region with a single peak, and the total area under the respective thermographs was assumed to be the total heat of the reaction for the respective reaction systems. For BB flour (10% and 15% addition levels), an exothermic peak corresponded to a continuing exothermic reaction with increase in temperature and it was also visible in the DSC scan. Higher BB content and the overall more viscous mixture results in a slightly earlier start of adhesive mixture curing, while the gluing of the veneers is sufficient [31]. The isothermal section was without a visible peak at the temperature of 105 °C. The determined curing temperatures were equal or lower compared with the pure UF resin [5,108]. In that case the reaction enthalpy (ΔH) should be simply proportional to the degree of conversion during the curing process. The endothermic reaction in the DSC curves after resin curing is related to vapor loss. The hydrolysis of the cured UF resin might have contributed to the endothermic phenomenon [109]. A single exothermic peak was observed for the cure of pure UF system as well as for all BB concentrations.

At the beginning of the recording, the apparent increase in signal is typical for each DSC recording which is proportional to the heating rate, the heat capacity of the pan and the weight of the sample. The endothermic peaks correspond to the evaporation of the water present. The exothermic peaks correspond to the cure of the UF resin. The reaction enthalpy decreased when more WF and BB was added in the curing system of the UF resin. As seen in Table 7, ΔH of the curing reaction of the adhesive mixture in case of more WF and BB were significantly lower than those of Ref10.

Bark decreases the curing enthalpy of UF resin through diffusion control and the change in the reaction phase of the curing system. Due to its high water absorptivity, the use of higher amount of BB in UF resin results in increased viscosity of adhesive mixture. Hence, UF resin will contain less water, that will lead to reduction of the diffusion and mobility of UF resin molecules. The curing reactions may be influenced by diffusion because the mobility of the molecules and their reactive groups decrease when the molecular weight increases which results in crosslinking occurred between molecules at the latter stages of the curing process [11,109,110,111,112]. Very high BB and WF concentrations should slow down the curing process of the samples. These results were also confirmed by [113]. In Ref20 and BB20 samples, where the amount of WF and BB strongly affected the viscosity, this mechanism caused a slight decrease in mechanical properties.

On the other hand, the absorption of water in the resin by the BB leads to a decrease in the hydrophilicity of the glue and significantly slows down evaporation of water during curing. This effect can be seen in DSC and TGA figures. This effect saved the energy, normally consumed during the evaporation of water which caused a momentary decrease in the temperature of the sample and slowed down the curing process. This phenomenon negates the slowing down the curing process of the samples mentioned above in case of BB10 and BB15. That is why the addition of 15% BB as a filler in UF resin seems to be the best option and should not be exceeded. In the case of 15% BB, the curing process was not inhibited and the peak temperature was not shifted to higher temperatures compared to Ref10.

The adhesive mixture with BB acts as a heterogeneous system, when the UF resin is mixed with bark. This conclusion corresponds with our previous research using scanning electron microscopy (SEM) [28]. This research showed, that bark particles in the UF adhesive mixture are functional, that they perform the role of the filler in the adhesive composition and that they create the necessary environment of the glue mixture for pressing the plywood. Besides that, no agglomerations of bark particles could be seen and the UF resin disperses on the surface of bark it results in some unreacted functional groups [28]. This effect is most visible in case of BB20. The reaction enthalpy decreased even more for mixture BB20 than with BB10 and BB15. It suggests that the curing reactions reached a lower final degree of conversion for the mixtures of UF resin with bark than for the reference samples. Thus, too many bark (BB20) may partly inhibit the curing reaction of UF resin. These results correspond with results of research [109,111]. In addition, the adhesion strength of plywood decreased as the viscosity increased by adding more WF.

The decrease of ΔH can be also explained by the presence of formaldehyde scavengers in the bark, like tannin and lignin. Tannins and lignin react with free formaldehyde in the adhesive system which results in decrease of free formaldehyde. Decreases in free formaldehyde causes another decrease in ΔH, because the degree of crosslinking of the cured resins, as well as the reactivity of the hardening reaction, depends on the availability of free formaldehyde in the system [114,115]. Results of this research are also consistent with research by [116] where authors investigated the effect of formaldehyde scavenger on cure characteristic of UF resin. The results showed, that reaction enthalpy rapidly decreases as the amounts of scavengers increased from 0 to 12%. The reduction in reaction enthalpy observed was accounted for lower energy needed for UF curing in samples with greater amounts of formaldehyde scavenger. This evidence indicates that curing of UF become more active when higher amounts of formaldehyde scavenger is introduced in samples, in other words higher degrees of UF curing would be achieved when the sample consisting of greater scavenger amount is cured under the same condition. These results were confirmed also by [117,118].

The smallest curve decrease (Figure 3) was recorded for the BB addition of 10%. This corresponded to the lowest mechanical strength values of the laboratory-produced plywood panels (MOR, MOE and shear strength). This suggests that the addition of 15% BB as a filler in UF resin for plywood manufacturing is more appropriate than the 10% BB addition. This was also confirmed by the lowest formaldehyde emission values, achieved at 15% BB content. This idea is similar to the considerations of Park et al. [49] who observed the identical phenomena in particleboard. The formaldehyde emission of particleboard bonded with the modified UF resin decreased with an increase in the scavenger concentration to some extent. In addition, Park et al. [49] stated that in order to keep the mechanical properties of the particleboard at the required level, the additions to the adhesive mixtures must be reasonable. MOR and MOE of BB 20% added were already lower than the mechanical properties of plywood samples, fabricated with the addition of 15% BB to UF adhesive mixtures. The tendency of decrease in mechanical properties with further addition of BB (20% and more) continued. These results are in accordance with [119]. Authors investigated the effect of wood used on the curing characteristics of UF adhesive mixtures and evaluated the effects of extracts from sixteen wood species on adhesive curing behaviors, proving that the curing rate of UF mixtures increased as the addition content increased and reached a maximum at a certain share.

#### 3.4.2. TGA and DTG

**Figure 4:**
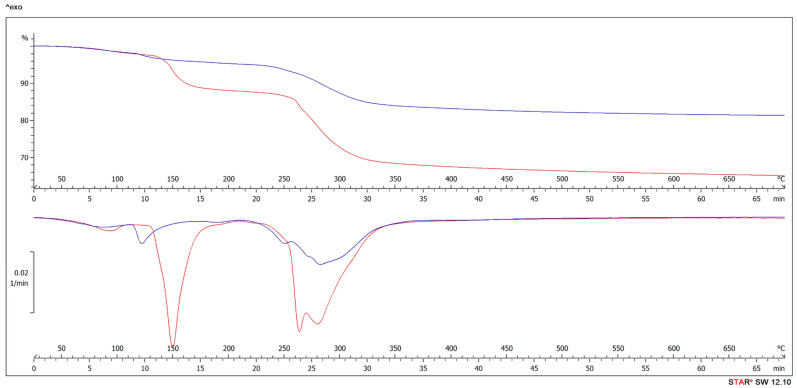
Thermal gravimetric analysis (TGA) curves (**top**) and derivative thermogravimetry (DTG) curves (**bottom**) of Ref10 (red) and BB15 (blue).

The effectiveness of BB as filler in UF resin for plywood manufacturing was also tested by TGA and DTG. Thermogravimetric analyses can be used for thermal stability and degradation analysis of the adhesive. The thermal behavior of Ref10 and BB15 adhesive mixture had three main stages (Figure 4).

In the first one, the so-called first-step degradation, corresponds to a loss of weight (until 15%). This mass lost is mostly caused by the evaporation of water and partial volatilization during polymer chemical modification to give progressively more stable structures [120,121]. Slow formaldehyde emissions at temperatures 100–200 °C results also in small mass loss. Above 250 °C there is the main polymer degradation with most weight loss due to the formaldehyde release, oligomer and water caused by the further cross-linking reaction between methylol groups [122]. Radicals formed by chain scission induce the formation of cyclic structures in the polymer which undergoes extensive fragmentation above 300 °C and at higher temperatures leaves a charred residue [120].

TGA and DTG analysis (Figure 4) showed, that BB addition to the adhesive mixture improves the UF thermal resistance in all temperature stages. In case of Ref10 adhesive mixture, significant weight loss was observed above 100 °C, mostly due to water evaporation. In case of BB samples, as the bark content increased, the water loss above 100 °C significantly decreased. As explained in the DSC part, the reason is that the BB absorbs some amount of water in the resin, thanks to its high wettability, which results in increase of viscosity and decrease the hydrophilicity of the glue. This procedure is also responsible for decrease in formaldehyde emission in case of BB samples, because the hydrolysis of UF resin under acidic and moisture conditions is known to be responsible for the formaldehyde emission from wood-based panels [123,124,125]. As seen in TGA curves, the main polymer degradation with almost 20% weight loss (Ref10) occurred from 250 °C to 350 °C. In case of the BB15 sample, the weight loss was significantly lower (about 10%). In case of BB15 sample, stabilized water in the bark increased the resistance of adhesive mixture to degradation. These results are in accordance with [123]. Authors showed that adding tannin (33 and 50%) to UF resin can significantly reduce the weight loss during curing resin at about 100 °C and the sequence of degradation changes with two steps instead of three.

**Figure 5:**
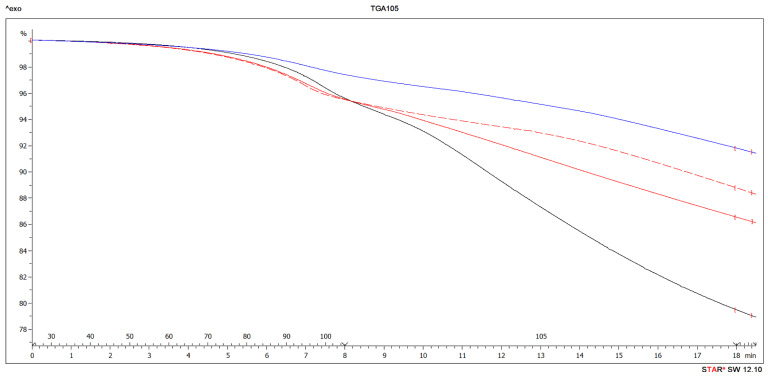
Left part: TGA curves to 105 °C of Ref10 (red dashed line), BB10 (red), BB15 (blue), BB20 (black). Right part: the isothermal section at the temperature of 105 °C.

Deeper analysis of TGA results showed, that during the isothermal section at temperature of 105 °C (Figure 5 right) the best results were achieved for BB15. The isothermal section was chosen to determine the behavior of the resin in case of extended time of pressing plywood during 105 °C. No other process (visible from DSC) was confirmed than drying of sample [126].

This is caused by most suitable concentration of soaked water, that is bounded in bark. In case of BB10 and Ref10, still high amount of water (explained in DSC and TGA part) is part of the resin and is continuously evaporated during isothermal heating at 105 °C. On the other hand, in case of BB20, there is very large amount of water soaked by bark which is not sufficiently bonded and evaporates quickly during isothermal heating at 105 °C [127]. The results of TGA and DTG analysis confirmed the results in DSC part, that the addition of 15% BB as a filler in UF resin is the best option and should not be exceeded.

## 4. Conclusions

Finely ground birch (*Betula verrucosa* Ehrhardt) bark can be effectively utilized as an eco-friendly alternative to wheat flour as a filler in UF adhesives for plywood manufacturing. The determined MOR, MOE, and shear strength values of the laboratory-produced plywood panels with 10% and 15% bark content were equal or higher than the mechanical properties of commercially produced plywood, and met the European standard requirements. A further increase in bark content to 20% resulted in slightly deteriorated MOR and MOE values. Markedly, the positive effect of BB addition in the UF adhesive mixture on the reduction of harmful formaldehyde emission in laboratory-fabricated plywood panels was also confirmed. The most significant decrease in formaldehyde release (up to 14%) was determined for the plywood sample produced with 15% BB. After four weeks, the decrease of formaldehyde was up to 51% in case of BB20. It can be concluded, that BB, considered as a wood processing industry waste or a by-product, has a significant potential to be used as filler in UF resins, providing an environmentally friendly, inexpensive solution for industrial valorization of bark as a bio-based formaldehyde scavenger. These results were also confirmed by DSC and TGA analyses performed and DTG curve.

## Figures and Tables

**Table 1:** Chemical composition of birch bark (BB) flour [62].

	BB (%)
Holocellulose (cellulose + hemicelluloses)	49.8
Lignin	27.9
Klason	26.4
Acid soluble	1.5
Suberin	5.9
Total extractives	17.6
Dichloromethane	5.1
Methanol	0.5
Ethanol	5.5
Water	5.2
Ash	2.9

**Table 2:** Carbohydrate composition of BB in % of total neutral monosaccharides [62].

	Glucose	Mannose	Galactose	Rhamnose	Xylose	Arabinose
BB	47.0	4.1	3.8	1.1	33.8	10.3

**Table 3:** Composition of adhesive mixtures used in this research work.

Adhesive Mixture.	Ref10	Ref20	BB10	BB15	BB20
Urea Formaldehyde (UF)	100 g	100 g	100 g	100 g	100 g
Adhesive Filler	10 g wheat flour	20 g wheat flour	10 g birch bark	15 g birch bark	20 g birch bark
NH_4_NO_3_ Hardener	10 g	10 g	10 g	10 g	10 g

**Table 4:** Average dynamic viscosity values (mPa·s).

Ref10	Ref20	BB10	BB15	BB20
1718.2 ±37.1	5362.6 ± 94.2	1058.4 ± 18.8	2878.4 ± 42.7	6427.8 ± 88.4

**Table 5:** Bending strength (MOR), and modulus of elasticity (MOE) values of plywood panels produced in this work.

Adhesive Composition	Bark Content, %	MOR (MPa)	MOE (MPa)
Ref10	0	118.4 ± 3.7 C	13,001.6 ± 306.3 C
Ref20	0	103.8 ± 4.0 A	11,827.5 ± 240.4 A
BB10	10	108.5 ± 2.2 B	12,380.4 ± 231.3 B
BB15	15	117.7 ± 2.0 C	12,934.5 ± 170.9 C
BB20	20	113.4 ± 3.2 BC	12,765.3 ± 193.5 BC

Different letters denote a significant difference. Means followed by the same letter do not statistically differ from each other (*p* ≤ 0.05).

**Table 6:** Shear strength of laboratory-produced plywood panels.

Adhesive Composition	Bark Content, %	Shear Strength (MPa)
Ref10	0	2.37 ± 0.09 C
Ref20	0	2.22 ± 0.14 B
BB10	10	2.11 ± 0.16 A
BB15	15	2.33 ± 0.12 C
BB20	20	2.39 ± 0.20 C

Different letters denote a significant difference. Means followed by the same letter do not statistically differ from each other (*p* ≤ 0.05).

**Table 7:** DSC results of adhesive compositions used in this work.

	Ref10	Ref20	BB10	BB15	BB20
*T*_onset_ °C	80.97	77.96	81.60	78.89	73.91
*T*_curing_ °C	88.04	86.61	89.09	89.32	88.32
ΔH J∙g^−1^	90.11	75.73	55.09	53.70	50.63

## Data Availability

The data presented in this study are available on request from the corresponding author.
